# Mucosal Exposure to Cigarette Components Induces Intestinal Inflammation and Alters Antimicrobial Response in Mice

**DOI:** 10.3389/fimmu.2019.02289

**Published:** 2019-09-25

**Authors:** Loni Berkowitz, Catalina Pardo-Roa, Geraldyne A. Salazar, Francisco Salazar-Echegarai, José P. Miranda, Gigliola Ramírez, José L. Chávez, Alexis M. Kalergis, Susan M. Bueno, Manuel Álvarez-Lobos

**Affiliations:** ^1^Departamento de Gastroenterología, Escuela de Medicina, Pontificia Universidad Católica de Chile, Santiago, Chile; ^2^Departamento de Genética Molecular y Microbiología, Facultad de Ciencias Biológicas, Millennium Institute on Immunology and Immunotherapy, Pontificia Universidad Católica de Chile, Santiago, Chile; ^3^Departamento de Nutrición, Diabetes y Metabolismo, Escuela de Medicina, Pontificia Universidad Católica de Chile, Santiago, Chile; ^4^Departamento de Endocrinología, Facultad de Medicina, Pontificia Universidad Católica de Chile, Santiago, Chile

**Keywords:** cigarette smoking, Paneth cells, Crohn's disease, inflammatory bowel disease, antimicrobial peptides, microbiota

## Abstract

The main environmental risk factor associated with the development of Crohn's disease (CD) is cigarette smoking. Although the mechanism is still unknown, some studies have shown that cigarette exposure affects the intestinal barrier of the small bowel. Among the factors that may be involved in this process are Paneth cells. These specialized epithelial cells are located into the small intestine, and they are able to secrete antimicrobial peptides, having an essential role in the control of the growth of microorganisms. Alterations in its function are associated with inflammatory processes, such as CD. To study how cigarette components impact ileum homeostasis and Paneth cells integrity, we used intragastric administration of cigarette smoke condensate (CSC) in mice. Our results showed that inflammation was triggered after mucosal exposure of CSC, which induced particular alterations in Paneth cells granules, antimicrobial peptide production, and a reduction of bactericidal capacity. In fact, exposure to CSC generated an imbalance in the fecal bacterial population and increased the susceptibility of mice to develop ileal damage in response to bacterial infection. Moreover, our results obtained in mice unable to produce interleukin 10 (IL-10^−/−^ mice) suggest that CSC treatment can induce a symptomatic enterocolitis with a pathological inflammation in genetically susceptible individuals.

## Introduction

Cigarette smoking has been associated as the major risk factor for several inflammatory gastrointestinal disorders, among them Crohn's disease (1). The impact of the toxic components of cigarette in the respiratory and vascular systems has been widely studied. However, how smoking affects the function of the gastrointestinal tract have not yet been clarified. Mucosal damage, alterations of mucosal immune response, and changes in gut irrigation are some of the proposed mechanisms that might explain the role of cigarette smoking in this disorder (2).

Accordingly, cigarette smoking is the main environmental risk factor associated with the development and progression of Crohn's disease (CD) (3). Although the pathogenesis of this disease is not completely understood, the evidence suggests that it would be the result of a complex interaction between genetic alterations, innate immune response, imbalance of intestinal microbiota, and environmental factors (4). Specifically, it has been postulated that after a certain stimulus, genetically-susceptible individuals develop an inadequate mucosal immune response against their intestinal microbiota. This response can lead to a pathological inflammation of the digestive tract, mainly involving the terminal ileum and the colon (5).

Interestingly, several studies show an association between cigarette smoking and the development of inflammation, specifically in the ileum, suggesting that the impact may be site-specific (6, 7). The particulate phase of cigarette smoke is composed mainly by low molecular weight components, such as nicotine, nitrosamines, polycyclic aromatic compounds, and heavy metals. The particulate phase can indeed be absorbed by epithelial cells of the body surfaces, such as the oronasal mucous membranes, skin, alveoli, and particularly through the gastrointestinal tract (1). As a matter of fact, high levels of nicotine, possibly the most studied particulate compound of cigarette, has been found in gastric fluids. These amount of nicotine could be 10 times higher than the levels found in arterial blood, and 80 times higher than the concentration observed in venous blood (8). Thus, the intestinal effects of cigarette smoking may be directly related to the amounts of particulate compounds swallowed that reach the intestine and could cause tissue damage.

Among the factors that may be involved in intestinal inflammation caused by cigarette smoking are Paneth cells (PCs). PCs are specialized epithelial cells of the small intestine that contain multiple secretory granules, filled with antimicrobial peptides and trophic factors, which are essential for the control microbial growth and maintaining intestinal homeostasis (9). Alterations in their function are associated with an imbalance of the normal microbiota, gastrointestinal infections, and inflammatory processes (10). Moreover, Paneth cells have been postulated as a site of origin for intestinal inflammation in ileal Crohn's disease, the location most commonly affected by the disease (11). However, the effect of cigarette smoking on Paneth cells has not been elucidated yet.

The aim of this study was to evaluate the impact of cigarette smoke particulate matter on the intestinal homeostasis and its bactericidal response, particularly on Paneth cells, using cigarette smoke condensate (CSC) in mice.

## Materials and Methods

### Ethics Statement

All the experiments that used mice were conducted in agreement with the international ethical standards and following the local animal protection guidelines. Experimental protocol No. 170329009 were reviewed and approved by the Scientific Ethical Committee for Research Safety and the Scientific Ethical Committee for Animal and Environment Care of the Pontificia Universidad Católica de Chile.

### Mice Strains

Seven-to eight-week-old C57BL/6 wild-type male mice (WT) and C57BL/6 IL-10^−/−^ mice (IL-10^−/−^) were originally purchased from Jackson Laboratories (Bar Harbor, ME, USA) and maintained in the pathogen-free animal facility at the Facultad de Ciencias Biológicas, Pontificia Universidad Católica de Chile. General condition and physiological score were evaluated and recorded every 2 days using a dedicated supervision protocol (Supplementary Table 1).

### Mouse Model of CSC Exposure

Mice received 200 μg CSC, 400 μg CSC or PBS by intraperitoneal or intragastric administration, 3 times a week for 2 weeks. CSC (Murty Pharmaceuticals, Lexington, KY, USA) is a commercial formula that contains total particulate matter prepared from a standard research cigarette (3R4F: University of Kentucky, KY, USA) and dissolved in DMSO. In each case (CSC treatment or vehicle), the total volume was 200 uL with a final concentration of 5% DMSO. According to the literature, these doses correspond to 8 and 16 cigarettes in a subject of average weight, based on the concentration of nicotine and the metabolic rate of rodents (12). For intragastric administration, mice were anesthetized using isoflurane (anesthesia was initiated with 5%, and maintained with 2% isoflurane). The intragastric administration (intragastric gavage) was done using a flexible cannula attached to a syringe and used to deliver the treatment directly into the stomach. One day after the last administration, mice were euthanized for organ recovery.

### Cotinine Levels

Blood samples were collected from the submandibular region 45 min and 24 h after the last administration of CSC. Total blood was maintained during 30 min at 37°C and then it was centrifugate at 2,000 rpm for 10 min to collect serum. Serum was stored at −80°C until use. Cotinine (nicotine metabolite) serum levels were determined by ELISA (Abnova, catalog no. KA2264), according to the manufacturer's recommendations.

### Tissue Treatment for Histological Procedures

Distal Ileum and colon were dissected and perfused with 2 mL sterile phosphate-buffered saline (PBS, pH 7.4), and then fixed in 10% formalin solution. Tissues were embedded in paraffin (Tissue Processor Leica ASP300), and transversal sections of 5 mm were adhered to positively charged glass slides, deparaffinized, and used for Hemathoxylin-eosin (H&E), alcian blue-PAS (AB-PAS), and immunofluorescent (IF) staining.

### Histopathology and Morphometrics Analysis

For hispathology review, sections were stained with H&E or AB-PAS (pH 2.5) by routine methods. The histological score was performed from 0 (non-inflamed) to 12 (highly inflamed), on transversal sections of terminal ileum, proximal colon and distal colon, according to Schultz et al. (13). Also, morphology and morphometrics were assessed in ileum sections stained with AB-PAS. At least 15 crypts and villi were assessed per section by a single blinded observer, analyzing 3–5 section per mouse containing full villi. Villus-crypt unit (VCU) length and the number of goblet cells per VCU were measured or counted at 10× magnification. The number of Paneth cells per crypt and granules organization was examined at 100× magnification under immersion oil. Then, Paneth cells were classified according to the organization of their granules as normal (D0), disorganized (D1), depleted (D2), and diffuse (D3) (11). Also, the number of intermediate cells (between Goblet and Paneth cells) was assessed and classified according to their location as: adjacent to PCs (P1), in the middle zone of proliferation (P2), or in upper positions over differentiated Goblet Cells (P3).

### Immunofluorescent Staining

After deparaffinization, antigen demasking was performed by boiling the tissue samples in 0.01 M Sodium Citrate Buffer (pH 6.0)—Tween 0.05% for 45 min. After cooling down the samples to RT, slides were washed 2 times with distilled H_2_O for 5 min each, and then 3 times in TBS-Tween 0.1% for 5 min each. Sections were blocked with 50 mL of TBS-Tween 0.1%—FBS 5%—BSA 10%, for 60 min at RT in a humidified chamber. Then, the slides were incubated overnight at 4°C with primary antibody against Lysozyme (goat anti- Lysozyme C 1:200, Santa Cruz catalog no. sc-27958) or primary antibody against RegIIIγ (rabbit anti- RegIIIγ 1:200, Abcam catalog no. ab198216). Fluorochrome-conjugated secondary antibodies, Donkey anti-goat IgG (H+L) Cross-Adsorbed Alexa Fluor 488 (Invitrogen, catalog no. A11055, diluted 1:200) and Donkey anti-rabbit IgG (H+L) Alexa Fluor 594 (Abcam, catalog no. ab150080, diluted 1:400), were used to incubated the slides for 1 h at RT in a humidified chamber. Sections were mounted using Vectashield Antifade Mounting Medium with DAPI (Vector Laboratories, catalog no. H-1200), and visualized using an epifluorescence microscope (Nikon Eclipse E200).

### Transmission Electron Microscope

Sections of the distal ileum (1 mm) were fixed for 16 h by immersion in 2.5% glutaraldehyde in cacodylate buffer 0.1 M pH 7, and then, washed tree times with cacodylate buffer 0.1 M pH 7. The sections were post-fixed with 1% osmium tetroxide (OsO_4_) for 90 min, and washed 3 times with bidistilled water. Then, sections were treated with 1% aqueous uranyl for 1 h, and sequentially dehydrated through graded acetones. Sections were left overnight in epon/acetone 1/1 and then in pure resin for 4 h. Finally, sections were included in fresh resin and polymerized in an oven at 60°C for 24 h. Ultrafine sections (80 nm) were obtained using an ultramicrotome Leica Ultracut R, which were incubated with 4% uranyl acetate in methanol for 1 min and lead citrate for 5 min. The grids were examined using a Phillips Tecnai 12 electron microscope operated at 80 kV at a magnification of 1700–2250X. Transmission electron microscopy was performed at the Advanced Microscopy Facility UC.

### Real Time PCR

The mRNA levels of *cryptdin-1, cryptdin-4, reg3*γ, and *lysozyme* were measured by quantitative real-time PCR analysis. Briefly, total RNA was extracted from distal ileum (whole tissue, 4 cm) using TRIZOL reagent (Invitrogen, catalog no. 15596026) according to the manufacturer's instructions, and then treated with DNase I Amplification Grade (Invitrogen, catalog no. 18068015) to eliminate DNA. A total of 1 μg RNA was reverse transcribed to cDNA using iScript RT Supermix (Biorad, catalog no. 1708890), according to the manufacturer's instructions. The resulting cDNA was amplified by real-time PCR in a StepOnePlus thermocycler (Applied Biosystems, CA) using the SsoAdvanced Universal SYBR Green Supermix (Biorad, catalog no. 1725270). The primers are listed in Supplementary Table 2. The amplification conditions were as follows: 30 s at 95°C and 40 cycles of 15 s at 98°C and 1 min of annealing and extension at 56°C. The relative quantification values were calculated using a comparative threshold cycle (2^−ΔΔct^) program on StepOne software, using *gapdh* as housekeeping gene.

### Bacterial Strains and Culture Conditions

*S*. Typhimurium ATCC14028 strain (STM) was originally obtained from American Type Culture Collection and *S*. Typhimurium 14028s ΔphoPQ::Kan (STMΔ*phoPQ*) was generated from ATCC14028 and both strains were kindly provided by Dr. Carlos Santiviago (Universidad de Chile, Santiago, Chile). Strains of *S*. Typhimurium aliquots were stored at −80°C in Luria-Bertani (LB) broth (tryptone 1%, yeast extract 0.5%, and NaCl 0.5%) supplemented with 20% glycerol. To perform infection assays, aliquots were grown with agitation at 37°C in LB broth until OD600 equal to 0.6 was reached. Then, bacterial doses were resuspended in sterile phosphate-buffered saline (PBS).

### Infection Protocol and Bacterial Load Quantification

After 48 h of the last CSC administration, a group of mice was anesthetized with isoflurane and infected with 1 × 10^5^ CFU of STM or 1 × 10^6^ CFU STM Δ*phoPQ* by intragastric gavage in 200 μl of PBS. Stool samples were collected at the first and second day post-infection, to perform bacterial counts. To evaluate bacterial load in organs, mice were euthanized 5 days post infection with STM or 2.5 days post infection with STM Δ*phoPQ*. Spleen, liver, blood and MLN were recovered from infected mice and the homogenized tissues were serially diluted in sterile PBS and seeded on LB or McConkey agar plates, in triplicates. Plates with LB solid medium supplemented or not kanamycin were used, depending on the strain. All colony forming units (CFUs) were quantified 24 h later and normalized based on organ weight. Bacterial growth in the plates was expressed as colony-forming units per organ, per mL of blood o per mg of feces.

### 16S Ribosomal RNA Gene Sequence Analysis of Fecal Microbiota

Fecal samples were collected in sterile plastic containers with 10% v/v DNA/RNA Shield™ (Zymo Research, catalog. no R1100-250) and stored at −80°C until further processing. Genomic DNA was extracted from 250 ul of stored stool using the ZymoBIOMICS™ DNA Mini Kit (Zymo Research, catalog. no D4300) according to the manufacturer's protocol. Fecal samples were collected from each group before and after treatment, under the same conditions. Only those differences that were not present at the initial time were reported.

Library preparation for the V3–V4 region was performed under the Illumina 16S Metagenomic Sequencing Library Preparation Protocol (Part # 15044223 Rev. B), using primers **319f** (5′-ACTCCTACGGGAGGCAGCAG-3′) and **806r** (5′-GGACTACHVGGGTWTCTAAT-3′) and sequenced on a MiSeq Illumina System (Illumina, San Diego, CA, USA), using the MiSeq Reagent kit V3 (Illumina, catalog no. MS-102-3003), with a 300 bp paired-end reads protocol. Raw sequence reads were generated and de-multiplexed by Theragen Etex Co, Ltd (Suwon, Korea). SRA files were deposited to NCBI database under Accession Number PRJNA525412.

Library adapters were trimmed using CutAdatp v1.11 (14) and paired-end reads merged using FLASH v1.2.11 (15). Reads with quality Q >20 and length >300 pb were kept using SICKLE v1.2.11 and chimeric sequences were discarded with ChimeraSlayer (16). The number of OTUs was determined by clustering the pre-processed sequences from each sample with a 97% identity cut-off using QIIME v1.8.0 (17). Taxonomic abundance was counted with RDP Classifier v2.11 (18).

Alpha diversity for each sample was determined using the Shannon index (19) and transformed to “effective number of species” (20). Beta diversity was measured according to Bray-Curtis distance (21). Principal component analysis (PCA) was then performed based on the measured beta diversities using Clustvis (22). For comparisons of relative abundances, the non-parametric Mann-Whitney *U*-test was used.

### Clinical Signs of Colitis

IL-10^−/−^ mice treated with CSC or vehicle were evaluated every 2 days and classified based on the onset of diarrhea, mucus in stools, perianal edema, or prolapse according the clinical score of colitis signs (described in Supplementary Table 1).

### Statistics Analysis

Statistical analyses were performed to analyze the data obtained on this work. Unpaired Student's *t*-test was employed to assess whether the means of two normally distributed groups differed significantly, and Mann Whitney *U*-test as its non-parametric counterpart. One-way ANOVA with Tukey's post-test was performed to compare multiple means. Two-way ANOVA with Bonferroni's multiple comparisons post-test was also used in some experiments. All statistical analyses were performed using Prism v6 software (GraphPad Software, San Diego, CA, USA).

## Results

### Intragastric CSC Exposure Causes Intestinal Inflammation

To evaluate the impact of cigarette components on the gastrointestinal mucosa, WT C57BL/6 mice received 200 or 400 μg of CSC by intragastric administration (i.g. CSC). To evaluate whether the intestinal impact depends on a systemic effect, another group of mice received equivalent doses of CSC by intraperitoneal administration (i.p. CSC). In both cases, we observed that there was absorption of the components of the cigarette, demonstrated by high levels of cotinine detected in serum of mice that received CSC (Supplementary Figure 1A). Indeed, the levels reached after 45 min of 400 μg of CSC administration were similar to those detected on an average smoker (23).

**Figure 1:**
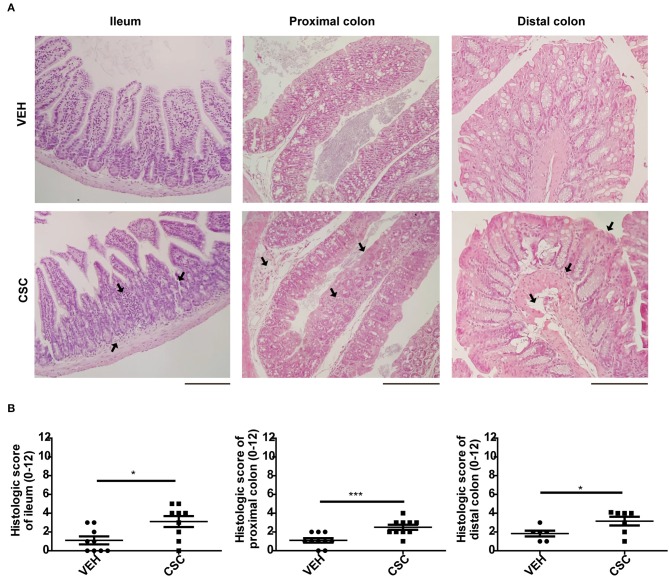
Intragastric CSC exposure causes intestinal damage. **(A)** Representative images of ileum, proximal colon, and distal colon sections stained with hematoxilin-eosin, of mice treated with 400 μg i.g. CSC or vehicle. **(B)** Histopathological analysis of ileum, proximal colon and distal colon of mice treated with 400 μg i.g. CSC or vehicle (*n* = 9, *t*-student, ^*^p < 0.05, ^***^p < 0.001). Scales bars are 200 μm. The same magnification was used for each section.

Histopathological analyzes showed that i.p. CSC treatment did not cause significant damage in the intestinal mucosa, as compared to the vehicle administration (Supplementary Figures 1B,C). However, administration of i.g. CSC caused a significant inflammation in the ileum (Supplementary Figure 1B). Moreover, the higher dose of i.g. CSC also generated histological alterations in the colon (Supplementary Figure 1C). This result was confirmed with a larger group of mice treated with 400 μg i.g. CSC, where the histopathology analyzes of the distal ileum, proximal colon and distal colon showed that i.g. CSC administration caused mild inflammatory infiltrate and distortion of the epithelial architecture, in all the areas evaluated (Figures 1A,B). Interestingly, in both ileum and colon sections, the i.g. CSC administration caused multifocal lesions, similar to those observed in Crohn's patients. According to the results obtained in these assays, we chose the treatment of 400 ug of i.g. CSC for subsequent analyzes of mucosal factors that may be involved in the inflammation observed in the gastrointestinal tract.

### Exposure to CSC Causes Morphometric Changes in the Ileal Epithelium

**Figure 2:**
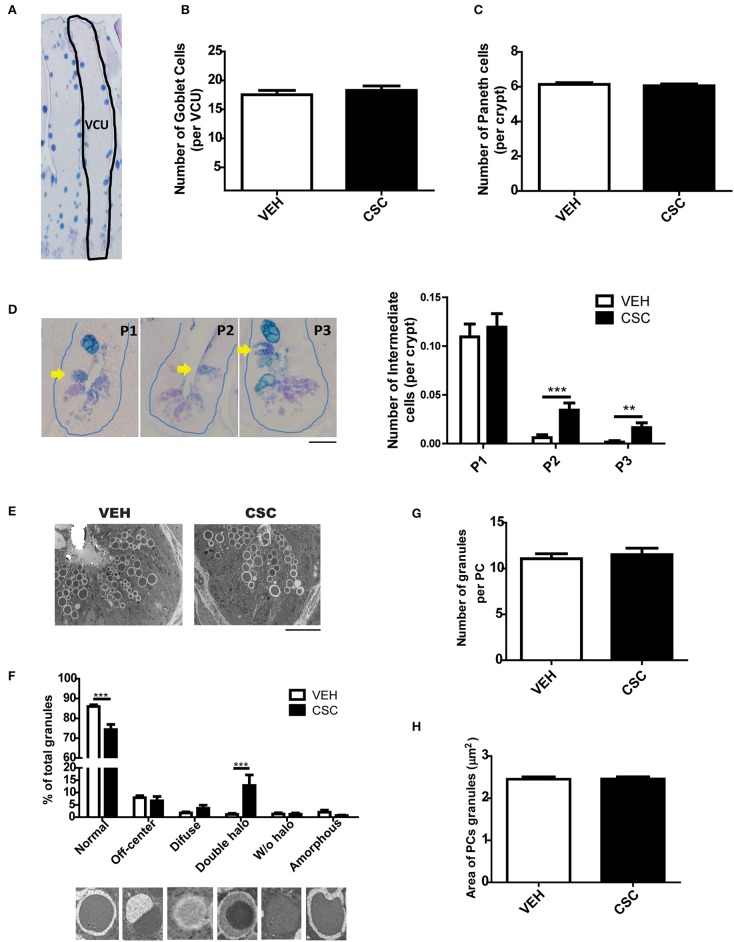
Exposure to CSC causes cellular anomalies in the crypts of Lieberkühn. **(A)** Representative image of ileum epithelium stained with AB-PAS. The dashed line denotes a villus-crypt unit (VCU). **(B)** Number of Goblet cells per VCU of mice treated with 400 μg i.g. CSC or vehicle (*n* = 6 mice). **(C)** Number of Paneth cells per crypt of mice treated with 400 μg i.g. CSC or vehicle (*n* = 6 mice). **(D)** Quantification of Intermediate Cells per crypt of both groups, according to their location, in: adjacent to Paneth cells (P1), in the middle zone of proliferation (P2), or in upper positions over differentiated Goblet Cells (P3) (2-way ANOVA, *post-hoc* Bonferroni ^**^p <0.01, ^***^p < 0.001, *n* = 660 crypts). Scale bar is 25 μm. **(E)** Representative electron micrograph of Liberkühn crypts of mice treated with 400 μg i.g. CSC or vehicle. Scale bar is 10μm. **(F)** Classification of granules, according to their TEM morphology in: normal, off-center, diffuse, double halo, without halo and amorphous (*n* = 3 mice, 2-way ANOVA, *post-hoc* Bonferroni ^***^p < 0.001). **(G)** Number of granules per Paneth cell (VEH *n* = 79 PCs, CSC *n* = 86 PCs). **(H)** External area of Paneth cells granules (VEH *n* = 79 PCs, CSC *n* = 86 PCs).

To evaluate in more detail the histological effects of CSC at ileal level, ileum sections of WT C57BL/6 mice were stained with alcian-blue PAS (AB-PAS) and morphometrics analysis were performed. We observed that the intestinal tissue of mice treated with CSC showed a reduction in villus-crypt length (Supplementary Figure 2A) consistent with the atrophic villi detected in the histopathological analysis of some animals with ileal inflammation. However, no differences were detected in the ileal perimeter (Supplementary Figure 2B), number of goblet cells per villus-crypt unit (Figures 2A,B), or in the number of Paneth cells per crypt (Figure 2C). Also, PCs were classified according to the organization of their granules as normal (D0), disorganized (D1), depleted (D2), and diffuse (D3), but no differences were observed between both groups (Supplementary Figures 2C,D).

Using AB-PAS, TEM, and immunofluorescence staining for lysozyme, it was possible to observe intermediate cells, which share characteristics between Goblet cells and Paneth cells (Figure 2D and Supplementary Figure 2E). These cells have been considered progenitor forms of secretory cells, originated from stem cells. Considering that they should be located in the proliferation zone of the crypt, we decided to classify them according to their location, in: adjacent to PCs (P1), in the middle zone of proliferation (P2), or in upper positions over differentiated Goblet Cells (P3). We observed that mice treated with CSC had a significantly higher number of intermediate cells outside the bottom of the crypt (Figure 2D), suggesting an increased requirement of epithelium regeneration or some alterations in the maturation of Paneth cells. Moreover, using TEM, it was possible to observe that mice treated with CSC presented a greater quantity of abnormal granules, with a semi-dense halo surrounding the electrodense core (Figures 2E,F). However, we did not observe differences in the number of granules per cell (Figure 2G) or in their size (Figure 2H). To assess whether the CSC generates death and regeneration of the epithelium of the crypts, we analyzed mitotic bodies and evidence of cell death by TEM. While we did not find signs of cell proliferation (mitotic bodies), we observed clear signs of cell damage and death, such as RE distention, degenerating granules, necrotic nucleus (Supplementary Figure 3).

### CSC Reduces the Expression of Antimicrobial Peptides

**Figure 3:**
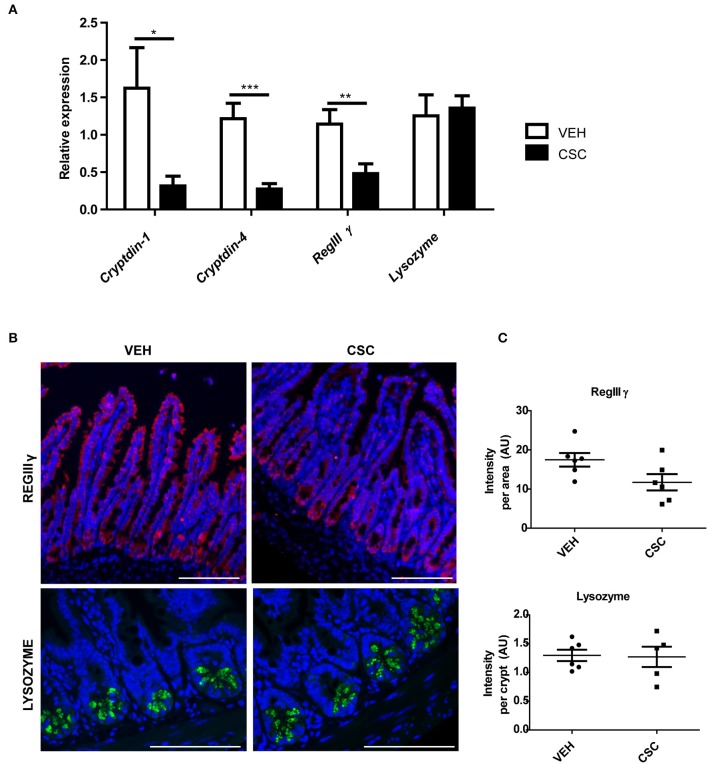
CSC reduces the expression of some antimicrobial peptides. **(A)** Relative expression of *cryptdin-1, cryptdin-4, regIII*γ, and *lysozyme* in ileum sections of mice treated with 400 μg i.g. CSC or vehicle (*n* = 9, *t*-student, ^*^p < 0.05, ^**^p < 0.01, ^***^p < 0.001). **(B)** Representative images of RegIIIγ immunodetection in ileal epithelium (upper panel) and Lysozyme in Lieberkühn crypts (lower panel). RegIIIγ in red, Lysozyme in green, and DAPI in blue. Scales bar are 100 μm. **(C)** Red fluorescence intensity normalized by the epithelial area (upper panel, *n* = 6, *t*-student *p* = 0.06) and Green fluorescence intensity normalized by the number of crypts (lower panel, *n* = 6, *t*-student *p* = 0.76).

Considering the differences observed in the ultrastructure of PCs granules, we wanted to evaluate whether CSC treatment also generates functional changes in these cells. To address this aim, we analyzed the impact of CSC in the transcription of some of the gene encoding main antimicrobial peptides present in murine PCs. We observed that CSC resulted in reduced production of the mRNA for *cryptdin-1, cryptdin-4*, and *regIII*γ, without affecting *lysozyme* mRNA production (Figure 3A).

In order to assess whether these changes also occurred at protein level, the presence of RegIIIγ and Lysozyme was evaluated by immunofluorescence in ileum sections (Figure 3B). We observed that RegIIIγ was present throughout the entire epithelium (Figure 3B). However, the sections showed a reduced immunodetection of RegIIIγ in the epithelium of the mice treated with CSC, as compared to those treated with vehicle. In fact, the main difference was observed in the villi, and not in the crypts (Figure 3B, yellow arrows). However, when we quantified the fluorescence intensity per tissue, these differences were not statistically significant (Figure 3C). On the other hand, consistently with the results obtained by qRT-PCR, lysozyme immunodetection showed no difference between CSC-treated and vehicle-treated mice. In both cases, the presence of these proteins was limited to the granules of the Paneth cells (Figure 3B), which showed similar fluorescence intensity per crypt (Figure 3C). These results demonstrate that intragastric exposure of CSC alters the ileal production of proteins required to control microbial proliferation in the intestine of mice.

### Exposure to CSC Increases the Susceptibility of Mice to Develop Ileal Inflammation Against Bacterial Infection

**Figure 4:**
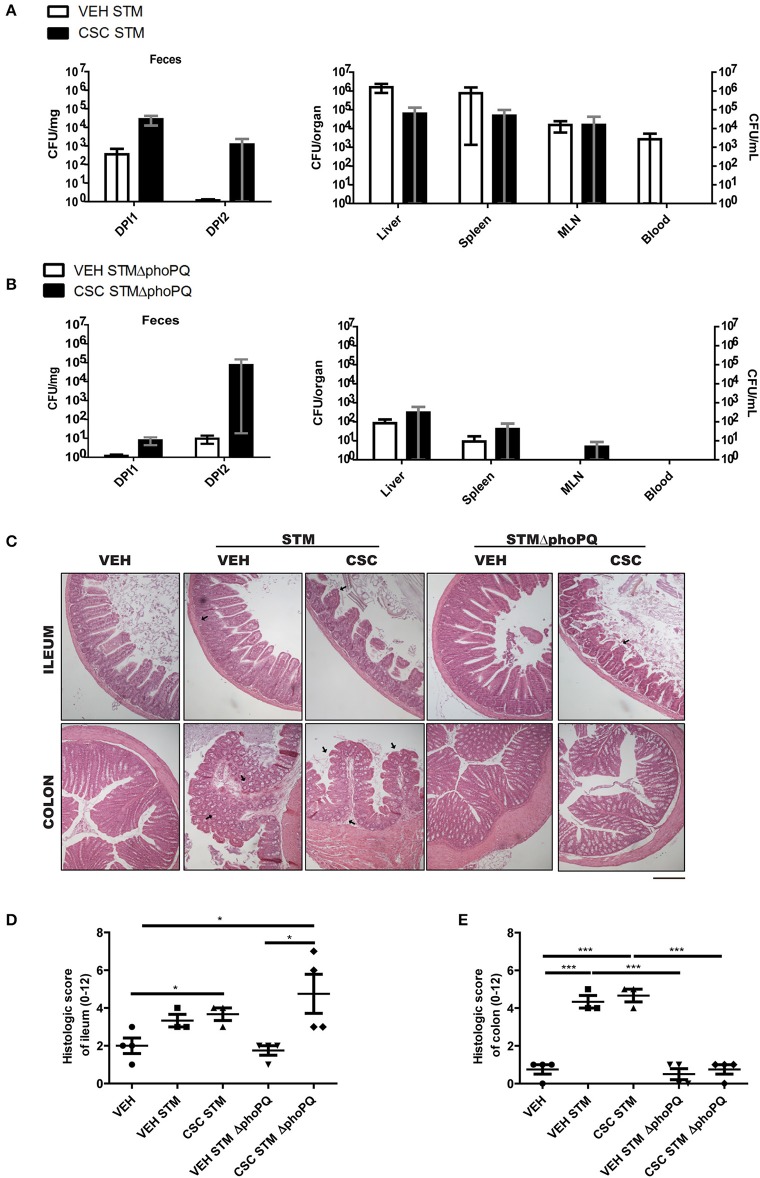
Exposure to CSC increases the susceptibility to respond in a pathological way against bacterial infection (*n* = 3 or 4). **(A)** Bacterial burden of mice infected with *S*. Typhimurium wild-type, pre-treated with CSC or vehicle. Left panel: Bacterial burden in heces evaluated at day 1 (DPI1) and 2 (DPI2) post-infection. Right panel: Bacterial burden in liver, spleen, mesenteric lymph nodes (MLN) and blood, evaluated at day 5 post-infection. **(B)** Bacterial burden of mice infected with *S*. Typhimurium Δ*phoPQ*, pre-treated with CSC or vehicle. Left panel: Bacterial burden in heces evaluated at day 1 (DPI1) and 2 (DPI2) post-infection. Right panel: Bacterial burden in liver, spleen, mesenteric lymph nodes (MLN) and blood, evaluated at day 2.5 post-infection. No significant differences were found (Mann-Whitney). **(C)** Representative images of ileum and colon sections stained with hematoxilin-eosin, of mice treated with CSC or vehicle and infected with *S*. Typhimurium wild-type or *S*. Typhimurium Δ*phoPQ*. Scale bar is 200 μm. **(D)** Histopathological analysis of ileum and **(E)** colon sections (ANOVA, Tukey *post-hoc* ^*^p < 0.05, ^***^p < 0.001).

To evaluate whether the impact of CSC on bactericidal peptides could increase the risk of gastrointestinal infections, WT mice pre-treated with CSC or vehicle were orally infected with *S*. Typhimurium 14028 (STM) or *S*. Typhimurium 14028s ΔphoPQ::Kan (STM Δ*phoPQ*). STM has the ability of colonize the intestine and generate a systemic infection (13, 24, 25). Consistently, bacterial loads in organs (Figure 4A) and intestinal inflammation (Figures 4C–E) was detected in all mice after infection with STM, but no differences in these parameters were detected between mice pre-treated with CSC and vehicle. Only an apparent increase in the fecal bacterial load of mice pre-treated with CSC was observed in comparison to the vehicle group (Figure 4A). Next, we evaluated the intestinal inflammation and bacterial dissemination in groups of mice infected with STM Δ*phoPQ*, which is an attenuated bacteria with a low ability to generate a systemic infection under normal conditions (26). The lack of a functional PhoPQ system makes this bacterium sensitive to antimicrobial peptides (27, 28). Therefore, its capacity to colonize capacity the intestinal epithelium will depend on the bactericidal capacity of the tissue. We observed that treatment with CSC did not increase STM Δ*phoPQ* translocation to germinal centers or bacterial load in organs (Figure 4B). Again, only an apparent increase was observed in fecal bacterial load of the mice pre-treated with CSC (Figure 4B). Interestingly, infection with STM Δ*phoPQ* generated inflammation in the ileum of mice pre-treated with CSC (Figures 4C–E), but not in the intestine of mice treated with vehicle. These results suggest that the ileal damage caused by the CSC makes these mice susceptible to develop inflammation after bacterial infections.

### Exposure to CSC Generates Changes in Fecal Bacterial Population

To evaluate the impact of CSC treatment on the gut microbiota of mice, stool samples were collected before and after the treatment with CSC or vehicle. To rule out any change in the microbiota due to genetic-environmental differences not attributable to CSC treatment, we only reported those differences found between CSC-treated and vehicle-treated mice at the end point.

**Figure 5:**
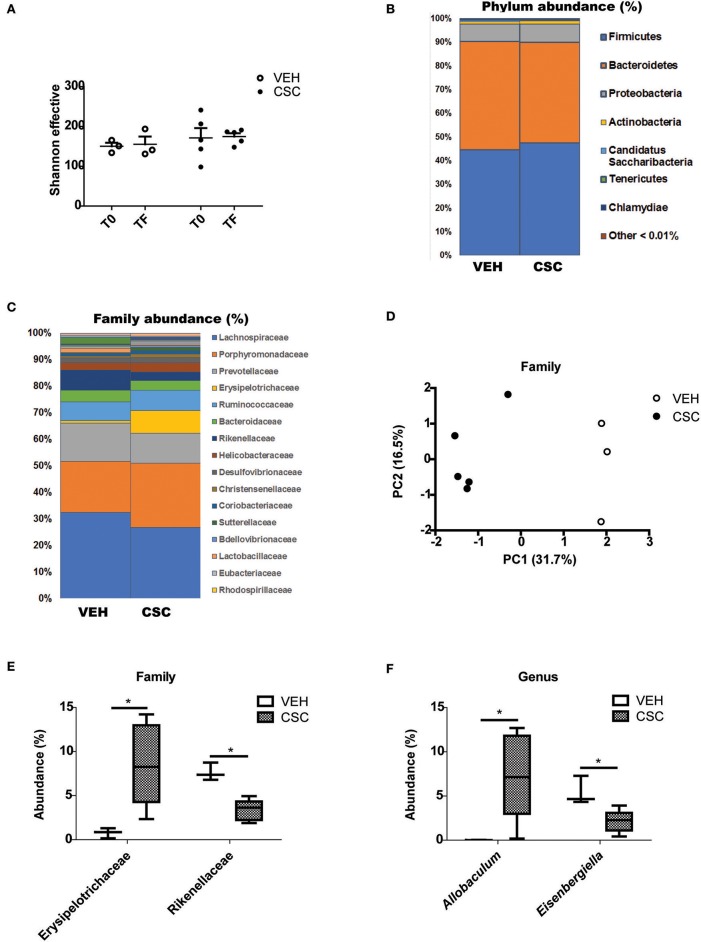
CSC generates an imbalance in the fecal microbial population. Sequence analysis of the 16S gene of rRNA from mice treated with vehicle (*n* = 3) or CSC (*n* = 5). **(A)** Effective number of Shannon species, evaluated before (T0) or after (TF) each treatment. **(B)** Relative percentage of dominant phylum detected in each group. **(C)** Relative percentage of dominant families detected in each group. **(D)** Principal Components Analysis (PC1 and PC2) of the β-diversity of phylum evaluated in fecal microbiota of mice treated with vehicle or CSC. **(E)** Relative percentage of families Erysipelotrichaceae and Rikenellaceae in mice treated with vehicle or CSC (Mann-Whitney, ^*^p < 0.05). **(F)** Relative percentage of genus *Allobaculum* and *Eisenbergiella* in mice treated with vehicle or CSC, at the final time (Mann-Whitney, ^*^ *p* < 0.05).

According to the results obtained, treatment with CSC did not generate significant changes in the microbial diversity of each sample (alpha diversity), evaluated as the Shannon index (Figure 5A). The analysis of relative abundance of the different Phylum, showed that the treatment with CSC did not generate significant changes in this taxonomic category (Figure 5B). However, it is possible to observe that the treatment with CSC significantly modified the relative abundance of some families in comparison to the vehicle (Figures 5C,D). Specifically, treatment with CSC generated an increase in Erysipelotrichaceae and a decrease in Rikenellaceae families (Figure 5E).

By comparing the relative abundance of the different genera, it was possible to observe a significant increase in the abundance of *Allobaculum* (Figure 5F). Its abundance after the treatment with CSC may explain the increase of Erysipelotrichaceae, the family to which *Allobaculum* belongs. Interestingly, a greater relative abundance of this genus has been previously associated with high levels of Cadmium (29), the most abundant heavy metal in the CSC. In the case of the Rikenellaceae family, no specific genus was altered. We also observed a significant reduction in the abundance of *Eisenbergiella* (Figure 5F), a genus belonging to the family *Lachnospiraceae*, known for its important capacity to produce butyrate (30). These results suggest that the direct exposure of the intestine to cigarette components generates an imbalance in the microbial population, which may contribute to the alteration of the intestinal homeostasis.

### CSC Induces Symptomatic Colitis in Susceptible Mice

Considering the intestinal inflammation caused by the exposure to CSC, it is feasible that this treatment could also stimulate the development of symptomatic colitis in susceptible individuals. To test this hypothesis we used IL-10-deficient (IL10^−/−^) mice, which spontaneously develop colitis depending on microbial exposure. IL-10^−/−^ were exposed to CSC in specific pathogen-free conditions, which induce IL-10^−/−^ mice to spontaneously develop local colitis and mild inflammation from the 8th week of age, as described in previous studies (31).

**Figure 6:**
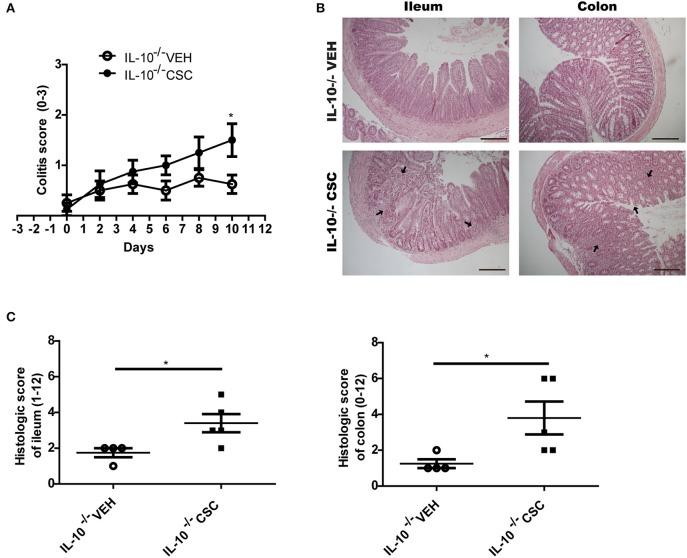
CSC induces symptomatic colitis in susceptible mice. **(A)** Colitis score of IL-10^−/−^ mice treated with vehicle or CSC (*n* = 8, 2-way ANOVA, post-Bonferroni ^*^p < 0.05). **(B)** Representative images of ileum and colon sections stained with hematoxilin-eosin, of IL-10^−/−^ mice treated with CSC or vehicle. Scale bars are 200 μm. **(C)** Histologic score of ileum and colon of IL-10^−/−^ mice treated with CSC or vehicle (*t*-student, ^*^p < 0.05, *n* = 4 o 5).

According to the clinical score of colitis signs recorded for each group, we observed that CSC treatment stimulated the appearance of signs of colitis in an accelerated and increased manner, as compared to the vehicle group (Figure 6A). The main signs observed were loose stools and perianal edema. In addition, these mice had a significantly higher histopathological score in the ileum and colon (Figures 6B,C). As in WT mice, the CSC administration caused multifocal lesions, similar to those observed in Crohn's patients. Furthermore, a group exposed to CSC was stimulated at the same time with conventional microbiota. However, it reached a severe inflammation within a few days after the treatment started (data not shown). This result suggests that both factors could act additively.

## Discussion

The results presented in this work show that intragastric exposure to the particulate matter of cigarette smoke generates intestinal inflammation and alteration of the antimicrobial response in mice, supporting the notion that cigarette smoke is a possible causal factor for Crohn's Disease. Interestingly, the damage generated by the treatment with CSC was substantially greater in response to intragastric administration than intraperitoneal administration. These results suggest that the effect depends on a local impact rather than a systemic one.

Cigarette smoking has been mainly associated with the development of ileal inflammation in humans (6). However, in our study we observed inflammation in both ileum and colon due to CSC treatment. These differences suggest that the impact of the cigarette is not necessarily site-specific. In fact, it could depend on the differential exposure of the cigarette components to the different segments of the intestine. In the case of humans, an important part of the particulate matter is swallowed by smokers (8). These components will reach the ileum slowly and chronically, being able to be absorbed mainly in this segment. In the case of the murine model described here, the intragastric administration of a bolus of CSC can stimulate gastric emptying and digestion, increasing the circulation of the treatment to more distal segments of the intestine. This could also explain the differences between our results and other studies in mice, where they only observe ileal inflammation after a long-term exposure to cigarette smoke in a whole-body chamber (7, 32).

After demonstrating the intestinal damage caused by the luminal arrival of CSC, we evaluated its effect on the bactericidal capacity of the intestinal mucosa. It has been reported that patients with ileal CD present a reduced expression of bactericidal peptides (33), and in addition, they have altered their intestinal microbiota (5). Specifically, we wanted to evaluate the impact of CSC on the integrity of Paneth cells, because they have been postulated as a possible site of origin of CD (11).

The results obtained in mice treated with CSC show that the recurrent arrival of components of cigarette smoke can damage the epithelium and alter the normal functionality of its cellular components, including Paneth cells. In fact, it is likely that cell damage caused by CSC does not concentrate on a single cell type, but rather encompasses several factors that participate in intestinal homeostasis. Particularly, in this study we observed that CSC effectively alters the bactericidal function of Paneth cells, evaluated as a reduced transcription of genes coding for cryptdins. However, we also observed a reduction in the bactericidal capacity of the whole epithelium, represented by a reduced expression of RegIIIγ in villi. In addition, we observed more intermediate cells in the proliferation zone of the Lieberkühn crypts in mice treated with CSC, and an increase in the percentage of immature granules in the Paneth cells. These results suggest that the damage caused by CSC stimulates a reparative process, which could be contributing to the functional alterations of the epithelium. In fact, it has been shown that after a chronic damage, Paneth cells can modify their transcriptional profile, expressing more markers to stimulate the proliferation of stem cells and the repair of the epithelium, reducing their bactericidal functions (34).

The impact of the cigarette on intestinal antimicrobial capacity incorporates in this scenario a third protagonist of the intestinal homeostasis: the intestinal microbiota. Our results suggest that the luminal arrival of CSC is capable of triggering an imbalance in the bacterial population, and in this way, contribute to the alteration of the normal homeostasis of the intestine. This could be explained by the reduced expression of the bactericidal peptides, but also by a possible direct effect of the luminal arrival of the CSC. For example, it was possible to observe a marked increase of the family Rysipelotrichaceae in mice treated with CSC. It has been described that this family is sensitive to α-defensins (35), and therefore the reduced production of bactericidal peptides due to CSC could contribute to their proliferation. In addition, the increased abundance of this family was mainly due to the increase of the genus *Allobaculum*. In fact, the most affected genus by the treatment with CSC was *Allobaculum*. It is reported that this genus increases in response to Cadmium (29), and therefore, the high abundance of Cadmium in the CSC (36) could directly explain this effect. On the other hand, we observed a significant decrease of the Rikenellaceae family in mice treated with CSC. These results are consistent with a study in which patients with CD and a decreased production of bactericidal peptides (due to a mutation in NOD2) presented a lower abundance of this family (37).

In addition, we observed that mice exposed to CSC are more susceptible to inflammation after an inoculation with a strain of *S*. Typhimurium that is sensitive to antimicrobial peptides. These results support the notion that CSC treatment could impair the intestinal bactericidal capacity, as a result of alterations in Paneth cells and the whole epithelium. This way, it contributes to the development of dysbiosis and increase the susceptibility to suffer gastrointestinal infections.

Previous studies have suggested that loss of intestinal homeostasis and increased contact with intestinal bacteria can trigger an exacerbated inflammatory response in this tissue (9). In fact, the results obtained in IL-10^−/−^ mice suggest that the loss of the intestinal homeostasis after CSC exposure can result in a symptomatic enterocolitis with a pathological inflammation in genetically susceptible individuals, as occurs in patients with CD. Even when IL-10^−/−^ mice spontaneously develop a colitis, sharing features with colonic IBD (31), various studies have support that the enterocolitis of these mice represents a model of CD. A study performed by Berg et al. has shown that inflammatory changes first appear in the colon of IL-10^−/−^ mice, but as the disease progresses, some mice also develop inflammation in the small intestine (38). Moreover, the enterocolitis exhibited by IL-10^−/−^ mice has been associated to an uncontrolled Th1 response that leads transmural lesions in aged mice (31, 38). In addition, recent results from our laboratory showed that IL-10^−/−^ mice have alterations in their Lieberkühn crypts even under SPF conditions, making them susceptible to an ileal noxa (39). This observation is consistent with a study describing that the exposure of IL-10 mice to cigarette smoke accelerated the development of colitis and increased the expression of interferon gamma in the small intestine (40).

In conclusion, the results obtained in this work support the participation of cigarette components in the loss of intestinal homeostasis and in the reduction of bactericidal capacity. Moreover, these results highlight once again the importance of the microbiota-host interaction in the origin of inflammatory pathologies, such as CD.

## Data Availability Statement

The datasets generated for this study can be found in SRA files were deposited to NCBI database, Accession Number PRJNA525412.

## Ethics Statement

All the experiments using mice were conducted in agreement with the ethical standards and according to the local animal protection law. All experimental protocols were reviewed and approved by the Scientific Ethical Committee for Animal and Environment Care and the Scientific Ethical Committee for Research Safety of the Pontificia Universidad Católica de Chile (Protocol #170329009). The committee declared that this project complies with the basic principles set forth in Chilean Law 20,380 on Animal Protection (2009), the Terrestrial Animal Health Code of the World Organization for Animal Health (OIE, 24th Edition, 2015), the European Directive 2010/63 / EU and the Guide for the Care and Use of Experimental Animals (NRC, 8th Edition, 2011), documents to which this institution ascribes.

## Author Contributions

All authors listed have made a substantial, direct, and intellectual contribution to the work and approved it for publication. Specifically, LB designed and performed the experiments. In addition, she wrote the manuscript with the help of SB and MÁ-L, LB, CP-R, and FS-E carried out the manipulation of the mice. GS and LB carried out the experiments with *S*. Typhimurium. JM analyzed the microbiota data. GR carried out the implementation of the immunofluorescence assays. JC participated in the staining and analysis of intestinal sections. CP-R analyzed the histological damage of the sections. AK contributed to the interpretation of the results and to the supervision of the project. SB and MÁ-L conceived the study and were in charge of overall direction and planning.

### Conflict of Interest

The authors declare that the research was conducted in the absence of any commercial or financial relationships that could be construed as a potential conflict of interest.
