# Supplementary material for: Mucosal Exposure to Cigarette Components Induces Intestinal Inflammation and Alters Antimicrobial Response in Mice
Source: Front Immunol. 2019 Sep 25;10:2289. doi: 10.3389/fimmu.2019.02289 (PMC6773925; doi:10.3389/fimmu.2019.02289)
Supplement: Supplementary file 1 [file Data_Sheet_1.pdf]

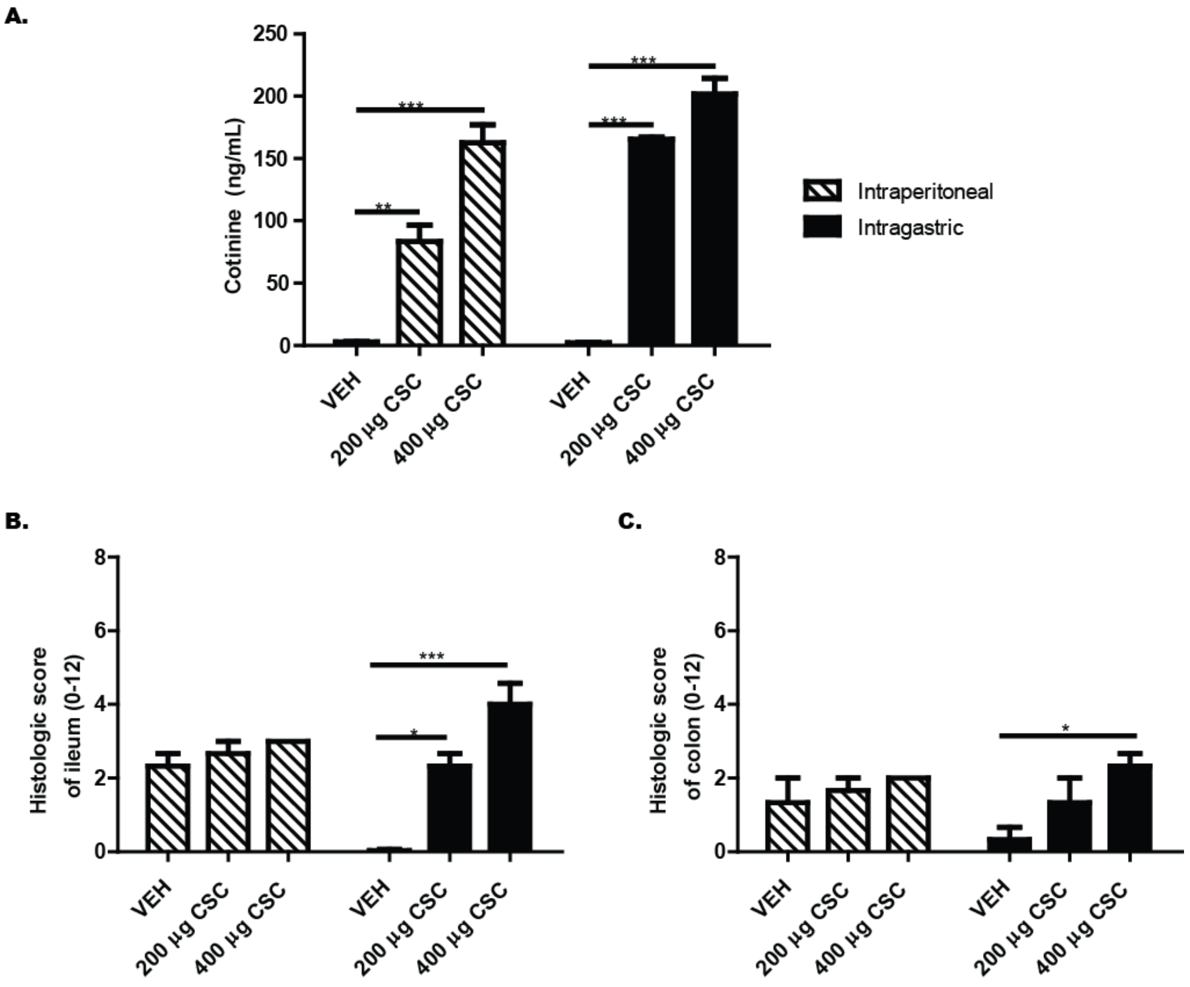


**Supplementary figure 1. Characterization of a murine model of exposure to CSC (n=3)**. A) Serum cotinine levels (ng/mL) determined 45 min after the last administration of CSC (200μg or 400μg) or vehicle, using intragastric or intraperitoneal administration (2-way ANOVA, post-hoc Bonferroni **p<0.01, ***p<0.001). B) Histopathological analysis of ileum sections (ANOVA, post-hoc Tukey *p<0.05, ***p <0.001). C) Histopathological analysis of colon sections (ANOVA, Tukey post-hoc * p<0.05).

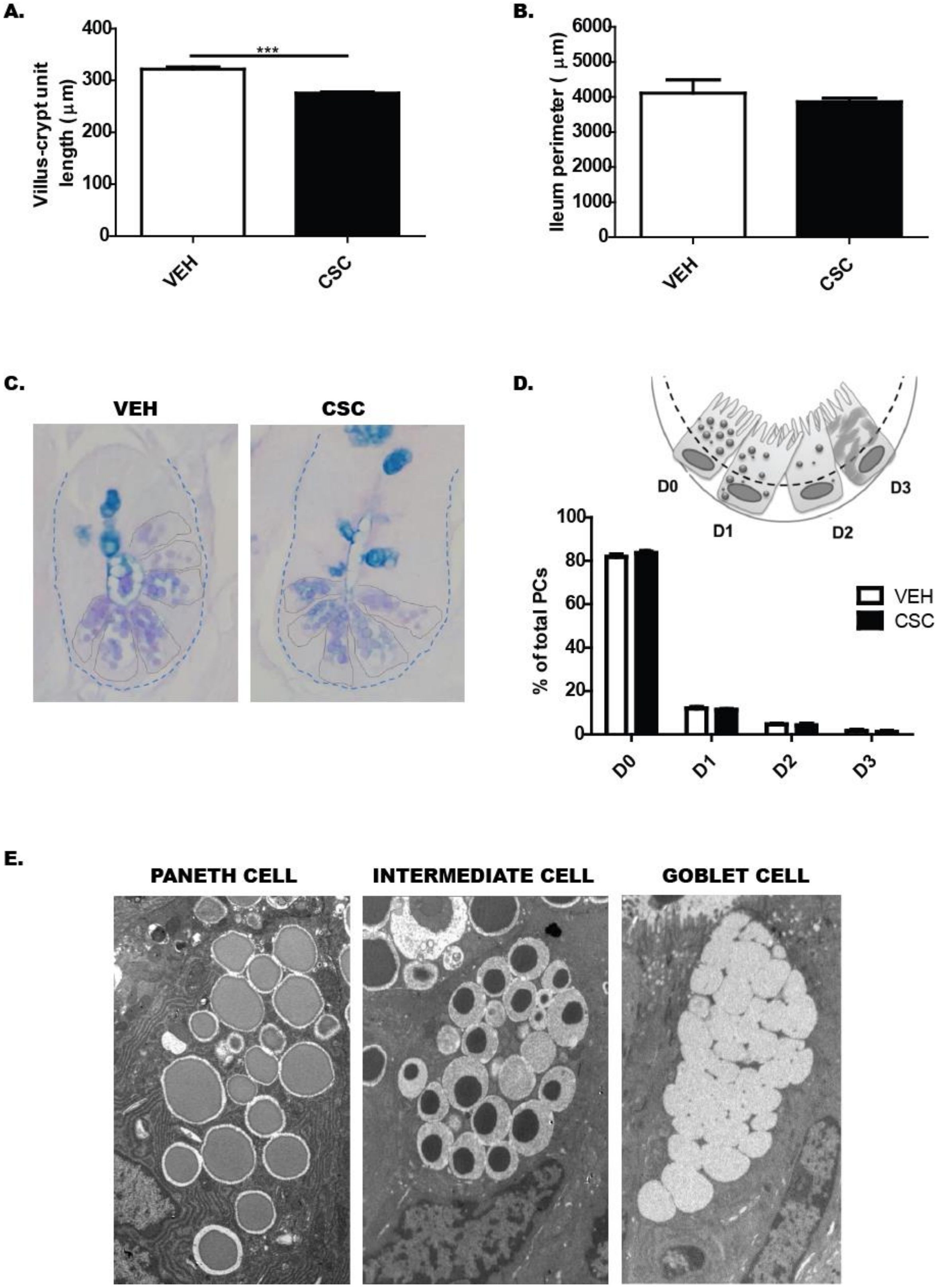
A.
Villus-crypt unit length (μm)
400
300
200
100
0
***
VEH
CSC
B.
Ileum perimeter ( μm)
6000
5000
4000
3000
2000
1000
0
VEH
CSC
C.
VEH
CSC
D.
D0
D1
D2
D3
% of total PCs
100
80
60
40
20
0
VEH
CSC
D0
D1
D2
D3
E.
PANETH CELL
INTERMEDIATE CELL
GOBLET CELL

**Supplementary figure 2. Morphometrics analysis of Lieberkühn crypts of mice treated with vehicle or CSC (n=6)**. A) Villus-crypt unit length (μm) (t-student, *** p<0,001). B) Ileum ileal perimeter (μm). C) Representative images of Lieberkühn crypts stained with AB-PAS, of mice treated with vehicle or CSC. D) Quantification of PCs according to the organization of their granules as normal (D0), disorganized (D1), depleted (D2) and diffuse (D3). E) Representative images of the granules of a Paneth cell, an intermediate cell and a Goblet cell, visualized by transmission electron microscopy.

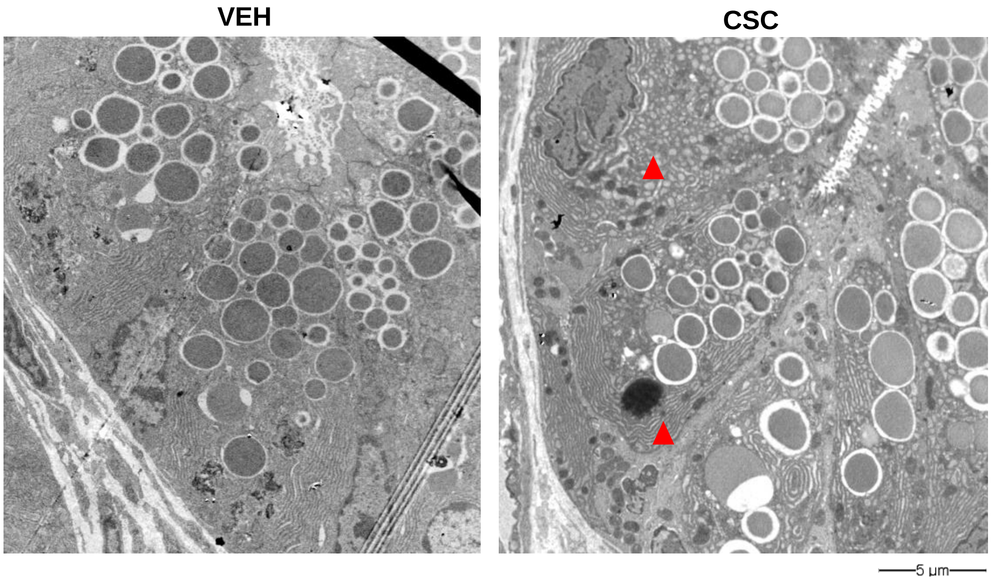


**Supplementary Figure 3**. Representative electron micrograph of Liberkühn crypts of mice treated with vehicle or 400µg i.g. CSC. Red arrows denote Paneth cells with distended endoplasmic reticulum, signs of damage and death.

**Supplementary Table 1. Clinical and physiological score.**

| Parameter | Observations | Score |
|---|---|---|
| **PHYSIOLOGICAL INDEX**<br>-Weight Loss<br>-Body temperature<br>(measure on abdominal surface) | Normal: No weight loss, Body temperature (T°) between 36,5-38,0°C. | 0 |
| | Mild: Weight lost less than 10%, T°=34,5°C-36,4°C | 1 |
| | Moderate: Weight loss between 10-20%, T°=34,5-36,4°C or 38,6-39.5°C | 2 |
| | Severe: Weight loss over 20%. T°<34,5 or >39.5°C | 3 |
| **GENERAL CONDITION** | Normal: Mice are active, moved around the cage, grooming, shiny hair. | 0 |
| | Mild: Reluctance to move, Restlessness, piloerection, rough hair, Porphyrin discharge (red-brown pigment around eyes and nostrils) | 1 |
| | Moderate: Weak attitude, hunched posture, dull or sluggish movements. | 2 |
| | Severe: Extreme weakness, mouse that does not move on the cage, sunken eyes and severe dehydration | 3 |
| **BEHAVIOR** | Normal: Alert attitude, grooming, eating, drinking and interacting with cage mates. | 0 |
| | Mild: Reduce grooming and movements into the cage, signs of tachypnea, remains into the bottom of cage. | 1 |
| | Moderate: Depression, lethargic, uncoordinated movements on cage, poor grooming, abdominal breathing. | 2 |
| | Severe: Extreme lethargy, prostration, panting, no grooming and fuzzy facial fur. | 3 |
| **COLITIS INDEX FOR IL-10 KO MICE** | Grade 0: Normal appearance of anus and feces. | 0 |
| | Grade 1: Mild inflammation of anus, perirectal edema, occasional tenesmus and feces with normal or soft consistency. | 1 |
| | Grade 2: Interment rectal prolapse, tenesmus, rectal edema, tenesmus feces with normal or soft consistency, mice need to be monitored more. | 2 |
| | Grade 3: Permanent prolapse, congestion and edema on rectal mucosa, diarrhea or absence of feces, blood on feces. Immediate euthanasia is advised. | 3 |

**DAI:** 0 - 3: Normal, 4 - 6: Careful monitoring, 7 a 9: Signs of severe pain, euthanasia would be considered, 10 a 12: Final Point criteria, Euthanasia would be performed.

**Supplementary Table 2. Oligonucleotide sequences used in qRT-PCR analysis.**

| | Sense | Antisense |
|---|---|---|
| *Cryptdin-1* | CTA GTC CTA CTC TTT GCC CT | TTG CAG CCT CTT GAT CTA CA |
| *Cryptdin-4* | GTC CAG GCT GAT CCT ATC CA | GGG GCA GCA GTA CAA AAA TC |
| *RegIIIγ* | TTC CTG TCC TCC ATG ATC AAA A | CAT CCA CCT CTG TTG GGT TCA |
| *Lysozyme* | CAA GAT CTA AGA ATG CCT GTG | TTC CGA ATA TAC TGG GAC AG |
| *GAPDH* | TGA AGC AGG CAT CTG AGG G | CGA AGG TGG AAG AGT GGG AG |
